# Ablation of Vitamin D Signaling in Cardiomyocytes Leads to Functional Impairment and Stimulation of Pro-Inflammatory and Pro-Fibrotic Gene Regulatory Networks in a Left Ventricular Hypertrophy Model in Mice

**DOI:** 10.3390/ijms25115929

**Published:** 2024-05-29

**Authors:** Ana Zupcic, Nejla Latic, Mhaned Oubounyt, Alice Ramesova, Geert Carmeliet, Jan Baumbach, Maria L. Elkjaer, Reinhold G. Erben

**Affiliations:** 1 Department of Biomedical Sciences, University of Veterinary Medicine, 1210 Vienna, Austria; azupcic1@gmail.com (A.Z.); nejla.latic@vetmeduni.ac.at (N.L.); alice.ramesova@vetmeduni.ac.at (A.R.); 2 Institute for Computational Systems Biology, University of Hamburg, Albert-Einstein-Ring 8-10, 22761 Hamburg, Germany; jan.baumbach@uni-hamburg.de (J.B.); maria.louise.elkjaer@uni-hamburg.de (M.L.E.); 3 Department of Chronic Diseases, Metabolism and Ageing, 3000 Leuven, Belgium; geert.carmeliet@kuleuven.be; 4 Ludwig Boltzmann Institute of Osteology, Heinrich-Collin-Strasse 30, 1140 Vienna, Austria

**Keywords:** vitamin D, vitamin D receptor, left ventricular hypertrophy, cardiomyocytes, spatial transcriptomics, inflammation, fibrosis

## Abstract

The association between vitamin D deficiency and cardiovascular disease remains a controversial issue. This study aimed to further elucidate the role of vitamin D signaling in the development of left ventricular (LV) hypertrophy and dysfunction. To ablate the vitamin D receptor (VDR) specifically in cardiomyocytes, VDR^fl/fl^ mice were crossed with Mlcv2-Cre mice. To induce LV hypertrophy experimentally by increasing cardiac afterload, transverse aortic constriction (TAC) was employed. Sham or TAC surgery was performed in 4-month-old, male, wild-type, VDR^fl/fl^, Mlcv2-Cre, and cardiomyocyte-specific VDR knockout (VDR^CM-KO^) mice. As expected, TAC induced profound LV hypertrophy and dysfunction, evidenced by echocardiography, aortic and cardiac catheterization, cardiac histology, and LV expression profiling 4 weeks post-surgery. Sham-operated mice showed no differences between genotypes. However, TAC VDR^CM-KO^ mice, while having comparable cardiomyocyte size and LV fibrosis to TAC VDR^fl/fl^ controls, exhibited reduced fractional shortening and ejection fraction as measured by echocardiography. Spatial transcriptomics of heart cryosections revealed more pronounced pro-inflammatory and pro-fibrotic gene regulatory networks in the stressed cardiac tissue niches of TAC VDR^CM-KO^ compared to VDR^fl/fl^ mice. Hence, our study supports the notion that vitamin D signaling in cardiomyocytes plays a protective role in the stressed heart.

## 1. Introduction

The increasing burden of cardiovascular diseases, including left ventricular hypertrophy (LVH), hypertension, coronary artery disease, and diabetes, places a significant strain on clinical resources and socioeconomic systems [1]. Despite remarkable progress in cardiovascular research and improved therapeutic strategies against major risk factors, morbidity and mortality due to heart failure complications are still a major concern.

Over the past three decades, a large number of clinical and preclinical studies have supported a role of vitamin D signaling in cardiovascular health. In this context, large epidemiological studies have presented strong evidence linking vitamin D deficiency with cardiovascular disease, even after adjusting for traditional cardiovascular risk factors [2,3,4]. However, it is still controversial whether vitamin D supplementation has a role in the therapy of cardiovascular diseases, as large intervention trials failed to show a beneficial effect on cardiovascular endpoints [5,6,7].

Apart from its well-established role in bone health and mineral metabolism [8,9], vitamin D signaling has been observed in many tissues expressing the vitamin D receptor (VDR), and it is thought that all direct regulatory actions of the vitamin D hormone, 1α,25-dihydroxyvitamin D_3_, are mediated via the VDR [10]. The expression of the VDR in the heart and in blood vessels suggests possible direct effects of vitamin D signaling in these tissues [10,11]. In the heart, the presence of the VDR has been demonstrated in cardiomyocytes close to T tubuli as well as in fibroblasts, and cardiac hypertrophy leads to an upregulation of VDR expression at the mRNA and protein levels [12,13]. Indeed, a plethora of preclinical studies employing different animal models has unveiled a multitude of mechanisms underlying the beneficial cardiovascular effects of vitamin D [14,15,16,17,18].

Nonetheless, inconsistencies in data, especially regarding the role of VDR signaling in cardiomyocytes, persist. While Li et al. [19] reported cardiomyocyte hypertrophy, hypertension, and upregulated renin secretion in global VDR knockout mice on a normal diet, we failed to find hypertension, increased renin secretion, or LVH in 3-month-old global VDR knockout mice on a rescue diet, which normalizes mineral homeostasis [20]. Chen et al. [21] reported that deletion of the VDR specifically in cardiomyocytes results in LVH under baseline conditions and upon induction with isoproterenol via the pro-hypertrophic calcineurin/NFAT/MCIP 1 signaling pathway. In contrast, we observed no difference in the hypertrophic response of wild-type (WT) mice and those lacking VDR globally subjected to transverse aortic constriction (TAC), a standard model of experimental LVH induced by chronically increased afterload [22]. In addition, we found no difference in heart function between WT mice and global VDR knockout mice after myocardial infarction in an earlier study [23].

A potential pitfall in studies addressing the cardiovascular role of vitamin D signaling is the pleiotropic effects of vitamin D in many different tissues, making it difficult to compare studies using global and conditional, tissue-specific VDR ablation models. The VDR is expressed ubiquitously, and approximately 3% of the human genome is directly regulated by vitamin D [10]. Hence, ablation of vitamin D signaling results in complex changes in global VDR knockout models.

The objective of this study was to gain further insight into the role of the VDR in cardiac hypertrophy and in LVH pathogenesis by specifically deleting the VDR in cardiomyocytes of mice and employing TAC as an experimental model of LVH induction. To address the complex effects of VDR signaling on gene regulatory networks in an unbiased, holistic manner, we performed spatial transcriptomics on cryosections of TAC hearts from mice with cardiomyocyte-specific deletion of the VDR and those of controls. We found that while LVH developed independently of cardiomyocyte VDR presence, cardiomyocyte-specific VDR deficiency was associated with LV functional impairment and more pronounced pro-inflammatory and pro-fibrotic gene regulatory networks in stressed cardiac tissue niches.

## 2. Results

### 2.1. Generation and Characterization of Cardiomyocyte-Specific VDR Knockout Mice

**Figure 1:**
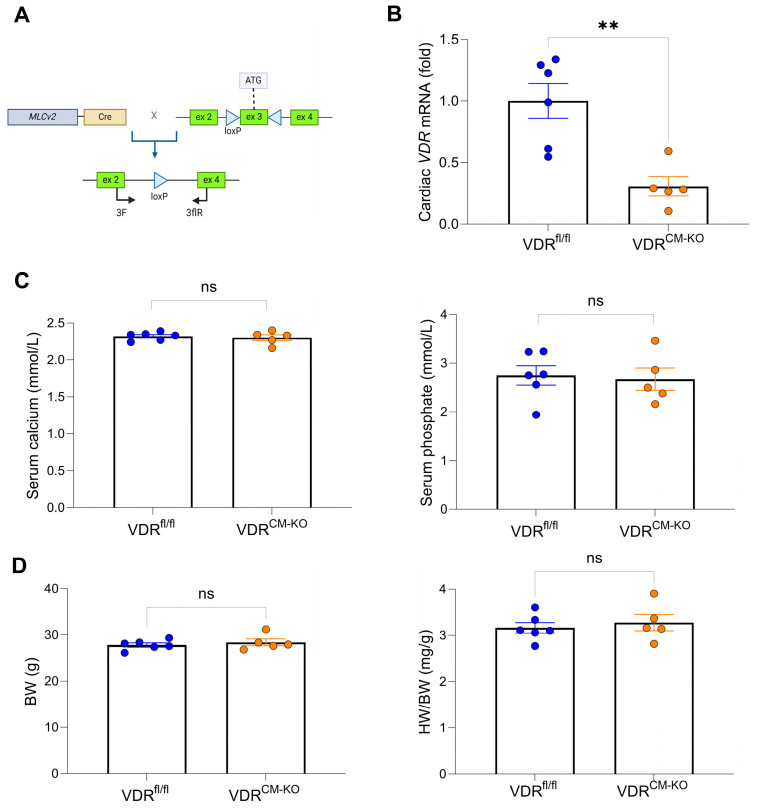
**Gene targeting strategy, cardiac ***VDR*** mRNA expression, as well as unaltered mineral metabolism and cardiac phenotype in VDR^CM-KO^ mice**. (**A**) Schematic illustration of Cre-LoxP strategy to generate cardiomyocyte-specific VDR knockout mice, showing the murine VDR allele with exons 1–3 and location of LoxP sites (triangles) surrounding exon 3. (**B**) Quantitative real-time PCR analysis reveals ~70% reduction of *VDR* mRNA levels at the whole heart level in VDR^CM-KO^ relative to VDR^fl/fl^ mice (n = 6 VDR^fl/fl^; n = 5 VDR^CM-KO^). (**C**) Serum concentration of calcium and phosphate (n = 6 VDR^fl/fl^; n = 5 VDR^CM-KO^) as well as (**D**) body weight and heart weight-to-body weight ratio (HW/BW) in VDR^CM-KO^ mice are comparable to those of VDR^fl/fl^ controls (n = 6 VDR^fl/fl^; n = 5 VDR^CM-KO^). Data are given as bar dot plots with SEM. ** *p* < 0.01 by unpaired *t*-test; ns, not significant. (**A**) was created with BioRender.com.

To decipher the effects of vitamin D deficiency on cardiac function, we generated a cardiomyocyte-specific VDR knockout mouse model using Cre-LoxP technology. Mice with a floxed *VDR* gene carrying loxP sequences surrounding exon 3 were previously created [24]. For specific deletion of the *VDR* gene in ventricular cardiomyocytes, we mated VDR^fl/fl^ mice with mice expressing Cre recombinase under the ventricle-specific Mlc2v promoter [25] (Figure 1A). The resulting genotypes included wild-type (WT), VDR^fl/fl^, Mlc2v^Cre/wt^, and VDR^fl/fl^/Mlc2v^Cre/wt^ mice, henceforth referred to as cardiomyocyte-specific VDR knockout mice (VDR^CM-KO^). We observed germline VDR deletion when the Cre allele was maternally inherited and therefore solely used male Cre-expressing mice for breeding in this study. To rule out effects of *Mlc2v* haploinsufficiency, we initially performed TAC experiments in wt/wt, wt/Cre^+^, and VDR^fl/fl^ mice. Wt/wt, wt/Cre^+^, and VDR^fl/fl^ mice showed comparable increases in systolic (SP) and pulse (PP) pressures as well as similar values for fractional shortening (FS) and ejection fraction (EF) after TAC (Supplementary Figure S1), corroborating earlier reports demonstrating that Mlc2v-Cre mice do not show an altered cardiac response to TAC-induced pressure overload compared with wt/wt mice [26].

The VDR^CM-KO^ mice were born at the anticipated Mendelian frequency without any gross phenotypic abnormalities. Recombination efficacy was confirmed by a ~70% reduction of cardiac *VDR* mRNA expression in VDR^CM-KO^ mice relative to controls (Figure 1B and Supplementary Table S1). This level of reduction in *VDR* mRNA expression is expected in VDR^CM-KO^ mice at the whole heart level because VDR expression is ubiquitous and other cell types, such as endothelial cells or fibroblasts, contribute to VDR expression in the heart. Serum levels of calcium and phosphate remained unaltered in VDR^CM-KO^ mice (Figure 1C). Moreover, VDR^CM-KO^ mice showed normal body weight and heart weight-to-body weight (HW/BW) ratio, relative to age-matched VDR^fl/fl^ controls (Figure 1D), suggesting that cardiomyocyte-specific VDR inactivation does not lead to a pathologic cardiac phenotype under standard resting conditions in 5-month-old male mice.

### 2.2. Development of Afterload-Induced Cardiac Hypertrophy Is Not Modulated by Loss of VDR in Cardiomyocytes

**Figure 2:**
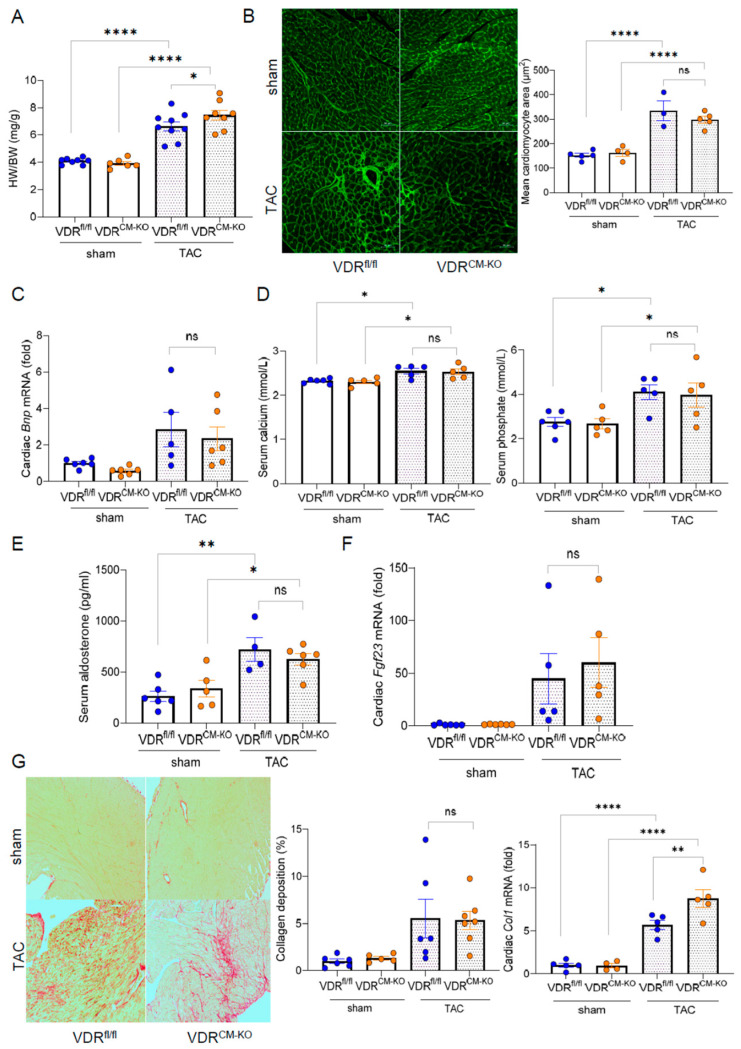
**Cardiomyocyte-specific VDR deletion does not aggravate left ventricular hypertrophy.** (**A**) Heart weight-to-body weight (HW/BW) ratio in sham-operated and TAC VDR^fl/fl^ and VDR^CM-KO^ mice (n = 8 VDR^fl/fl^ sham; n = 6 VDR^CM-KO^ sham; n = 9 VDR^fl/fl^ TAC; n = 8 VDR^CM-KO^ TAC). (**B**) Representative histological images of FITC-WGA staining and quantification of cardiomyocyte size reveal similar cardiomyocyte hypertrophy between the genotypes post-TAC (n = 5 VDR^fl/fl^ sham; n = 4 VDR^CM-KO^sham; n = 3 VDR^fl/fl^ TAC; n = 5 VDR^CM-KO^ TAC). Scale bar = 50 µm. (**C**) Relative mRNA expression of brain natriuretic peptide (*BNP*) is unchanged between TAC VDR^fl/fl^ and VDR^CM-KO^ mice (n = 6 VDR^fl/fl^ sham; n = 6 VDR^CM-KO^ sham; n = 5 VDR^fl/fl^ TAC; n = 6 VDR^CM-KO^ TAC). (**D**) Serum calcium and phosphate (n = 6 VDR^fl/fl^ sham; n = 6 VDR^CM-KO^ sham; n = 5 VDR^fl/fl^ TAC; n = 5 VDR^CM-KO^ TAC), and (**E**) aldosterone concentrations in sham-operated and TAC VDR^fl/fl^ and VDR^CM-KO^ mice (n = 6 VDR^fl/fl^ sham; n = 5 VDR^CM-KO^ sham; n = 4 VDR^fl/fl^ TAC; n = 6 VDR^CM-KO^ TAC). (**F**) Relative cardiac *Fgf23* mRNA expression (n = 6 VDR^fl/fl^ sham; n = 6 VDR^CM-KO^ sham; n = 5 VDR^fl/fl^ TAC; n = 5 VDR^CM-KO^ TAC) and (**G**) histological images of cardiac paraffin sections stained with picrosirius red (PSR) and quantification of PSR-stained area (n = 6 VDR^fl/fl^ sham; n = 5 VDR^CM-KO^ sham; n = 5 VDR^fl/fl^ TAC; n = 7 VDR^CM-KO^ TAC) as well as relative cardiac collagen 1 (*Col1a1*) mRNA expression in sham-operated and TAC VDR^fl/fl^ and VDR^CM-KO^ mice (n = 5 VDR^fl/fl^ sham; n = 4 VDR^CM-KO^ sham; n = 5 VDR^fl/fl^ TAC; n = 5 VDR^CM-KO^ TAC). Scale bar =100 µm. Data are given as bar dot plots with SEM. * *p* < 0.05, ** *p* < 0.01, **** *p* < 0.0001 by one-way ANOVA followed by Student–Newman–Keuls multiple comparison test; ns, not significant.

To examine the tissue-specific effects of vitamin D signaling in LVH, we employed TAC, a pressure overload–induced cardiac hypertrophy model. In line with previous studies, which show heart failure with reduced ejection fraction (HFrEF) development after 4 weeks of TAC [27], we terminated our experiments at this timepoint. TAC significantly increased the HW/BW ratio compared to sham-operated animals in all genotypes (Figure 2A). Based on previous findings [21], we hypothesized that loss of VDR in cardiomyocytes could accelerate cardiac pathology when subjected to pressure overload. Despite a slight increase in HW/BW ratio observed in TAC VDR^CM-KO^ compared to TAC VDR^fl/fl^ mice (Figure 2A), histological examination by FITC-labeled wheat germ agglutinin (WGA) staining revealed a similar increase in mean cardiomyocyte cross-sectional area post-TAC (Figure 2B). This indicates that cardiomyocytes respond to the afterload-induced hypertrophic stimulus independent of VDR. In addition, cardiac mRNA expression of the hypertrophy marker brain natriuretic peptide (*BNP*) was comparably increased after TAC in VDR^fl/fl^ and VDR^CM-KO^ mice (Figure 2C). Moreover, TAC mice were characterized by hyperphosphatemia, hypercalcemia, and increased serum aldosterone concentrations, independent of genotype (Figure 2D,E). It is well known that TAC is associated with an upregulation of cardiac mRNA expression of fibroblast growth factor-23 (FGF23) [22,28]. In line with these earlier reports, we observed approximately 50-fold upregulation of cardiac *Fgf23* transcription in response to TAC in both genotypes, with no differences between the genotypes (Figure 2F). We also investigated collagen remodeling and cardiac fibrosis, common LVH accompaniments, using picrosirius red (PSR) staining of paraffin sections. TAC-induced LVH was associated with a profound increase in interstitial fibrosis (Figure 2G). However, PSR staining did not demonstrate differences in cardiac fibrosis between TAC VDR^CM-KO^ and VDR^fl/fl^ mice (Figure 2G). In contrast, collagen type I (*Col1a1*) mRNA expression was upregulated in TAC VDR^CM-KO^ mice, relative to TAC VDR^fl/fl^ controls (Figure 2G). Nonetheless, as shown in the left and upper right panels of Figure 2G, this increase in *Col1a1* transcription in TAC VDR^CM-KO^ mice did not result in increased fibrosis as evidenced by PSR staining at 4 weeks post-TAC. Collectively, our data suggest that ablation of VDR expression in cardiomyocytes does not modulate afterload-induced cardiac hypertrophy, but results in higher collagen 1 levels at the transcriptional, but not at the histological level.

### 2.3. Lack of VDR Signaling in Cardiomyocytes Aggravates TAC-Induced LV Functional Impairment

**Figure 3:**
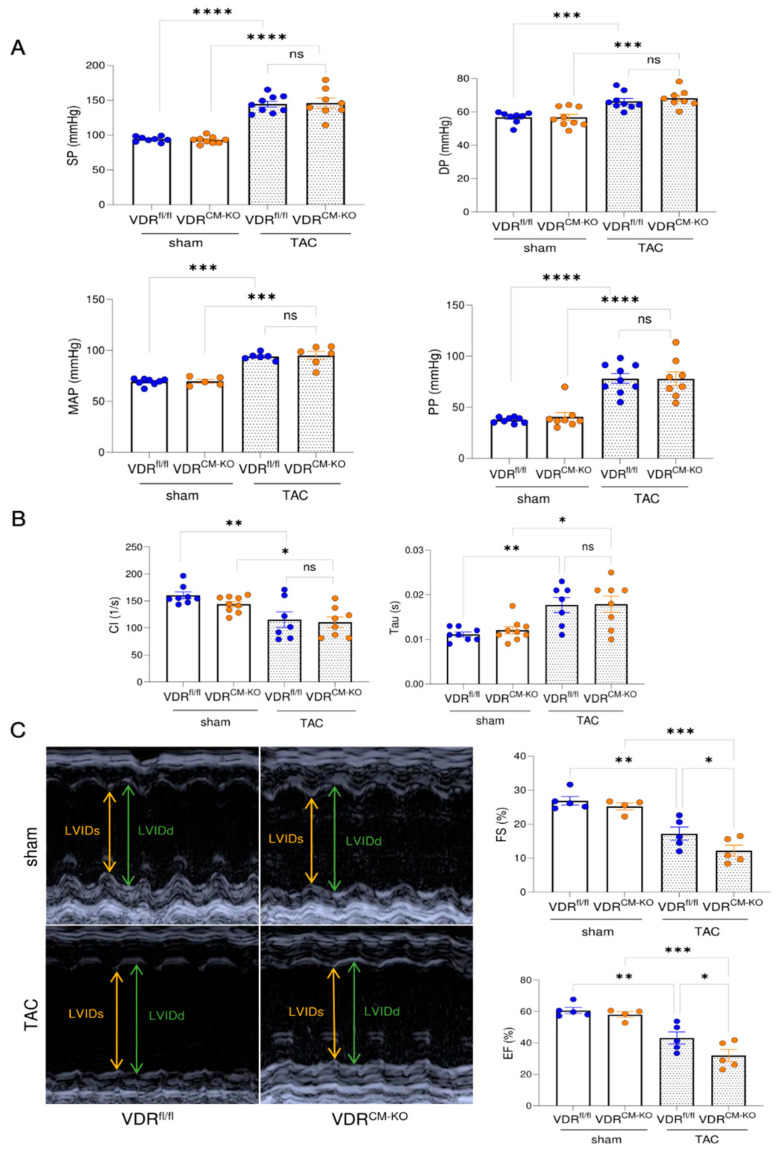
**Mice lacking VDR in cardiomyocytes display a more pronounced reduction in cardiac functionality after TAC.** (**A**) Systolic pressure (SP), diastolic pressure (DP), mean arterial pressure (MAP), and pulse pressure (PP) are comparably increased in VDR^fl/fl^ and VDR^CM-KO^ mice following TAC (SP, DP, PP: n = 8 VDR^fl/fl^ sham; n = 9 VDR^CM-KO^ sham; n = 9 VDR^fl/fl^ TAC; n = 8 VDR^CM-KO^ TAC; MAP: n = 8 VDR^fl/fl^ sham; n = 5 VDR^CM-KO^ sham; n = 6 VDR^fl/fl^ TAC; n = 6 VDR^CM-KO^ TAC). (**B**) LV contractility index (CI) and relaxation time (Tau) does not differ between TAC VDR^CM-KO^ and VDR^fl/fl^ mice (n = 8 VDR^fl/fl^ sham; n = 9 VDR^CM-KO^ sham; n = 7 VDR^fl/fl^ TAC; n = 8 VDR^CM-KO^ TAC). (**C**) Representative original echocardiograms in parasternal short axis M-mode (left). Parameters of cardiac functionality fractional shortening (FS) and ejection fraction (EF) (right) are significantly reduced in TAC VDR^CM-KO^ mice in comparison to TAC VDR^fl/fl^ controls (n = 5 VDR^fl/fl^ sham; n = 4 VDR^CM-KO^ sham; n = 5 VDR^fl/fl^ TAC; n = 5 VDR^CM-KO^ TAC). Data are given as bar dot plots with SEM. * *p* < 0.05, ** *p* < 0.01, *** *p* < 0.001, **** *p* < 0.0001 by one-way ANOVA followed by Student–Newman–Keuls post-hoc test. LVIDd and LVIDs, left ventricular internal diameter in diastole and systole; ns, not significant.

To assess cardiac functionality in our experimental model, we performed intra-arterial and intra-cardiac catheterization as well as echocardiography. TAC results in hypertension upstream of the constriction site, thereby increasing LV afterload. Lack of VDR in cardiomyocytes did not influence the TAC-induced increase in arterial systolic pressure (SP), diastolic pressure (DP), mean arterial pressure (MAP), and pulse pressure (PP) (Figure 3A). In addition, both TAC VDR^fl/fl^ and VDR^CM-KO^ mice developed elevated end diastolic pressure (EDP) relative to sham controls (Supplementary Figure S2A). Left ventricular catheterization showed a comparable decline in cardiac contractility and increase of relaxation time constant (Tau) in both TAC VDR^fl/fl^ and VDR^CM-KO^ mice, relative to sham controls (Figure 3B). In contrast, echocardiography revealed a further reduction of ejection fraction (EF) and fractional shortening (FS) in TAC VDR^CM-KO^ mice, relative to TAC VDR^fl/fl^ controls, suggesting a more pronounced development of HFrEF in mice lacking VDR in cardiomyocytes (Figure 3C). LV internal diameter in diastole and systole (LVIDd and LVIDs) tended to be elevated in TAC VDR^CM-KO^ mice in comparison with TAC VDR^fl/fl^ mice (Supplementary Figure S2B). However, the difference did not reach statistical significance. Taken together, these data suggest that the absence of VDR signaling in cardiomyocytes augments the TAC-induced reduction in cardiac functionality.

### 2.4. Spatial Transcriptomics Reveal More Pronounced Pro-Inflammatory and Pro-Fibrotic Gene Regulatory Networks in Hypertrophic Cardiac Tissue Niches of TAC VDR^CM-KO^ Mice

**Figure 4:**
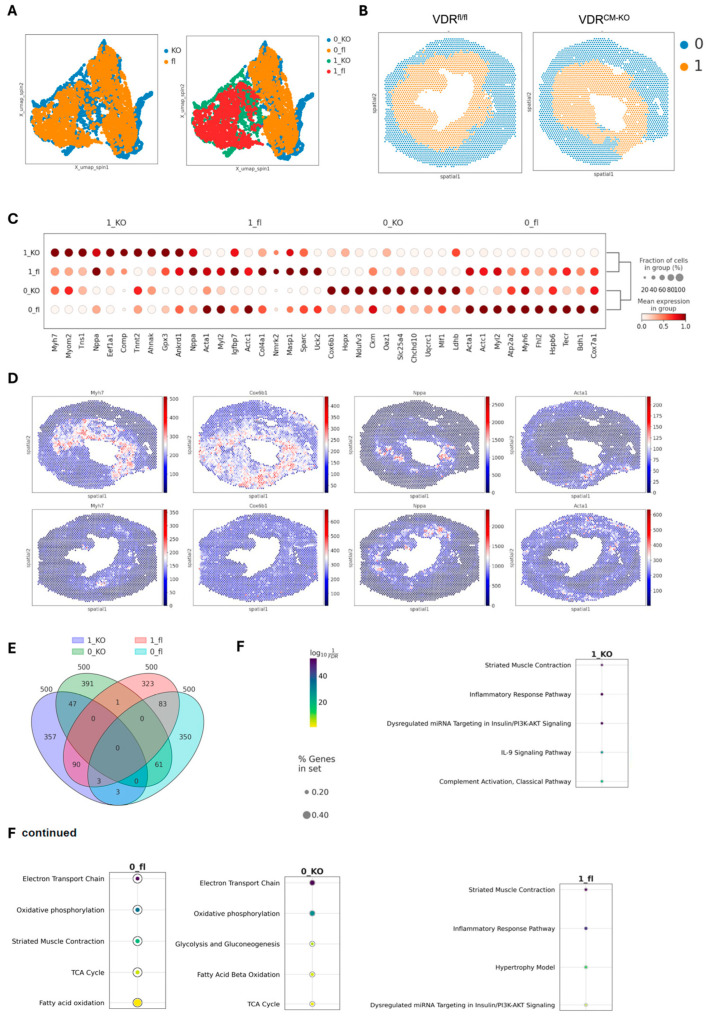
**Spatial transcriptomic analysis in the TAC-induced heart failure model in VDR^CM-KO^ and VDR^fl/fl^ mice.** (**A**) UMAP visualization of the integrated transcriptomic spots from cryosections of VDR^CM-KO^ and VDR^fl/fl^ TAC mice, showing similarities and differences in gene expression profiles. (**B**) Spatial mapping of clusters in heart sections of VDR^CM-KO^ and VDR^fl/fl^ TAC mice, revealing the cluster distribution. (**C**) Dot plot of the top 10 differentially expressed genes (DEG) identified across the combined clusters and conditions, highlighting key genes driving the distinction between clusters. (**D**) Visualization of spatial expression patterns for the most significant DEG identified per cluster (1_KO, 0_KO, 1_fl, 0_fl). (**E**) Venn diagram summarizing the overlap and uniqueness among the top 500 DEG across all combined regions and conditions. (**F**) GO (Gene Ontology) enrichment analysis for the top 500 DEGs, examining enriched pathways across the four identified clusters. KO, VDR^CM-KO^; fl, VDR^fl/fl^.

To gain further insight into the molecular changes induced by lacking VDR signaling in cardiomyocytes in our TAC model, we performed spatial transcriptomics on heart cryosections, focusing on the comparison between TAC VDR^fl/fl^ and VDR^CM-KO^ mice. The cross-sections were taken in the middle region of the ventricle. In agreement with profoundly reduced VDR expression in cardiomyocytes of VDR^CM-KO^ mice, we found only one spot with detectable VDR expression in the VDR^CM-KO^ mouse, whereas 44 spots were detected in the VDR^fL/fl^ mouse (Supplementary Figure S3). In all spots with VDR expression, we also detected cardiomyocyte-specific *Myl2* expression in the ventricle of the VDR^fl/fl^ mouse. Using the SPIN algorithm [29], we identified two distinct region clusters across TAC VDR^fl/fl^ and VDR^CM-KO^ samples, based on unique gene marker patterns that define specific tissue regions (Figure 4A). This spatial integration revealed two primary clusters corresponding to inner and outer tissue regions (cluster 0 representing the outer region and cluster 1 the inner region), with an even distribution of spots among them (0_fl (1649), 0_KO (1641), 1_fl (1414), and 1_KO (1327) (Figure 4B). The ‘outer’ region includes the outer LV wall and the right ventricle, whereas the ‘inner’ region includes the LV inner wall and septum (Figure 4B). For simplicity, we will refer to the two clusters as ‘inner’ and ‘outer’ regions henceforth.

The differential gene expression analysis identified the top ten signature genes distinguishing these clusters, with hierarchical clustering showing more similarities within regions than between genotypes (Figure 4C). The outer regions, characterized by genes such as *Myh6*, *Cox6b1*, *Ckm*, and *Acta1*, reflect typical heart function, underscoring their roles in maintaining cardiac physiology [30,31,32] (Figure 4C). Conversely, the inner regions showed elevated levels of myosin heavy chain-7 (*Myh7)* and ANP (*Nppa*), markers linked to cardiac stress and hypertrophy, similar to findings from single-cell RNA-seq studies in the same heart failure TAC model [33,34] (Figure 4D). The upregulation of *Myh7* has been linked to a transition from hypertrophy to heart failure, marked by an increase in *Myh7* mRNA and protein levels, particularly pronounced in severe cardiac stress [33]. Moreover, the variability in *Myh7* and *Nppa* expression among cardiomyocytes indicated a heterogeneous cellular response to pressure overload [34]. Noteworthy is the differential expression pattern of *Myh7* and *Myh6* encoding for β-myosin heavy chain (MHC) and α-MHC, respectively, representing distinct MHC isoforms critical for cardiac function [35,36,37]. In the inner, more stressed heart regions in TAC mice, *Myh7’s* presence correlates with its slower, more energy-efficient contractions, a beneficial adaptation during cardiac stress [36]. Conversely, *Myh6* predominates in the outer, more healthy areas, facilitating rapid contractions and higher cardiac output, though at greater energy expense [35]. This differential expression highlights the heart’s adaptability, with *Myh7* optimizing energy use under stress and *Myh6* enabling swift responses in normal conditions.

A detailed examination of the top 500 differentially expressed genes revealed distinct gene sets for each cluster, with minimal overlap, indicating unique molecular profiles (Figure 4E). GO (Gene Ontology) enrichment analysis further differentiated these regions, associating outer regions with normal heart function pathways, while inner regions showed enrichment for inflammatory pathways, with the VDR^fl/fl^ heart identified as the hypertrophy model (Figure 4F). The IL-9 and complement pathways specific for the inner region in the VDR^CM-KO^ heart hints at vitamin D’s regulatory impact on immune responses [38] in the hypertrophic heart (Figure 4F).

**Figure 5:**
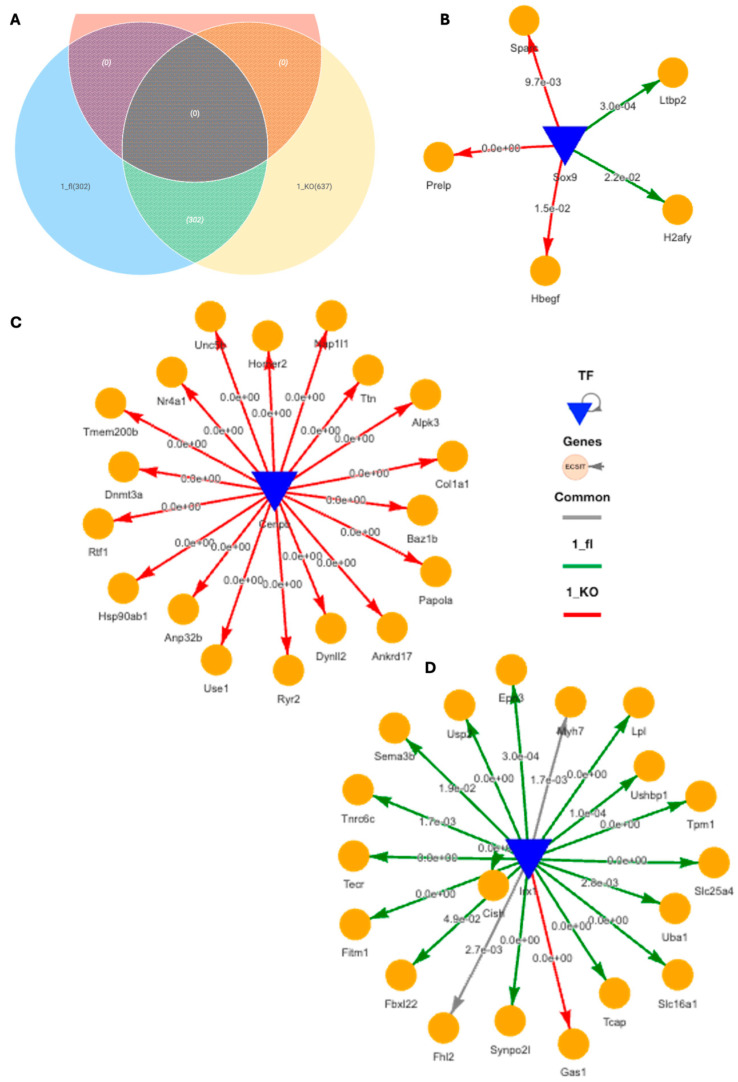
**Differential gene regulatory networks in cardiac hypertrophic tissue niche in TAC VDR^CM-KO^ and VDR^fl/fl^ mice.** (**A**) Venn diagram of shared and unique regulatory edges within the inner, stressed heart tissue region in TAC VDR^fl/fl^ and VDR^CM-KO^ mice. (**B**) SOX9-driven gene regulatory network (GRN) with target genes specifically in the VDR^CM-KO^ (1_KO, red) or in the VDR^fl/fl^ (1_fl, green) mouse. (**C**) CEBPB-driven GRN exclusively in 1_KO. (**D**) IRX1-driven GRN primarily in 1_fl. TF, transcription factor.

Using DiNiro [39] to analyze differential regulatory disease mechanisms between VDR^CM-KO^ (1_KO) and VDR^fl/fl^ TAC mice (1_fl) in the inner, more stressed hypertrophic tissue niche, we uncovered 302 shared edges, with an additional 302 unique to 1_fl and 637 unique to 1_KO (Figure 5A). Among the significantly highly ranked gene regulatory networks (GRNs), the Sox9-driven network stands out due to its impact in cardiomyocyte-driven hypertrophic cascades [40]. The target genes of Sox9 for the 1_KO are associated with extracellular matrix (ECM) and growth factor signaling (Sparc Prelp, Hbegf) indicative of fibrotic responses, while in 1_fl the targets (H2afy, Ltbp2) hint at mechanisms potentially protective against excessive fibrosis, involving genes in chromatin remodeling and TGF-beta regulation (Figure 5B). CEBPB-driven GRN exclusively regulate genes in 1_KO, such as Col1a1 (fibrosis) and Hsp90ab1 (stress response), as well as dynl2 and dbn3a (cytoskeletal changes), suggesting a focus on structural and stress adaptations (Figure 5C). Additionally, IRX1, a transcription factor associated with anti-fibrotic functions in myocardial health [41], predominantly regulates genes in 1_fl including Fhl2 (modulating cardiac hypertrophy) [42], Lpl (crucial for lipid metabolism and energy utilization) [43], Uba1 (key in protein degradation and cardiac stress management) [44], and Usp2 (regulation of overload-induced cardiac remodeling) [45] (Figure 5D). IRX1 is also the potential mechanistic regulator behind Myh7 for both 1_KO and 1_fl in the inner tissue niche (Figure 5D).

**Figure 6:**
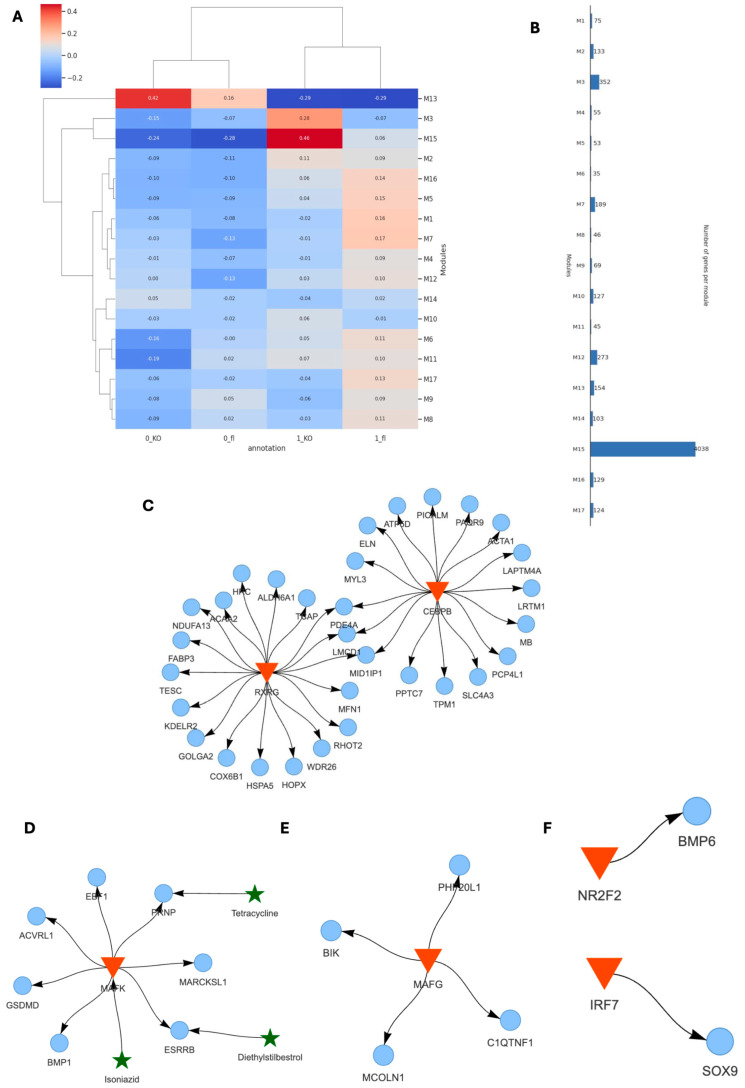
**Gene regulatory networks in different cardiac tissue niches in the absence of VDR.** (**A**) Overview of co-expression gene modules identified in VDR^CM-KO^ and VDR^fl/fl^ models across inner and outer heart regions, illustrating distinct co-expression patterns. (**B**) Comparison of co-expression gene module sizes. (**C**) GRN of module M13 in the outer tissue niche in the VDR^CM-KO^ heart (0_KO) including transcription factors (red triangles) and their target genes (blue circles). (**D**–**F**) Small GRNs in the inner hypertrophic tissue niche in the VDR^CM-KO^ heart (1_KO) with interacting repurposing drugs (green stars).

Using SCANet, we analyzed co-expression gene modules across inner and outer tissue regions of TAC VDR^CM-KO^ and VDR^fl/fl^ mice (0_fl, 0_KO, 1_fl, 1_KO) (Figure 6A). This approach enabled de novo identification of genes exhibiting synchronized expression patterns, thus facilitating an unbiased examination of distinct behavioral patterns and their functional implications within specific tissue niches. Notably, modules M13 and M15 showed a stronger correlation in 0_KO and 1_KO, respectively (Figure 6A). Module M13 contains 154 genes, whereas M15 consists of 4038 genes (Figure 6B). Within the 0_KO outer niche, the M13 module featured the major transcription factors RXRG and CEBPB, which targeted the outer signature gene markers (Cox6b1, Acta1) (Figure 4C and Figure 6C). Conversely, the M15 module in the inner niche of 1_KO revealed smaller GRNs, driven by, among others, the pro-inflammatory transcription factors Mafk and Mafg, targeting genes such as *C1qtnf1* (complement factor), *Acvrl1* (TGF-beta signaling for vascular integrity and fibrosis), *Bmp1* (ECM organization, impacting fibrosis and tissue repair), *Bik* (pro-apoptotic), *Mcoln1* (lysosomal function and autophagy), *Esrrb* (energy metabolism and mitochondrial function), and *Prnnp* (protective against oxidative stress) (Figure 6D,E). In addition, the transcription factor NR2F2 was connected to BMP6. Given that NR2F2 binding is very flexible, including normally regulating VDR through competitive binding [46], our finding of NR2F2 regulating BMP6 in VDR^CM-KO^ tissue may be a strategic shift in response to VDR’s absence (Figure 6E). Finally, Sox9, the potential regulator of cardiomyocyte-driven hypertrophic pathways (Figure 5B), is regulated by the pro-inflammatory transcription factor IR7 in KO_1 (Figure 6F).

## 3. Discussion

Taken together, our data indicate that vitamin D signaling in cardiomyocytes is dispensable for heart function under physiological conditions in 5-month-old mice. Additionally, we found that the development of cardiomyocyte hypertrophy in response to chronic pressure overload is independent of VDR expression in cardiomyocytes. However, lack of vitamin D signaling in cardiomyocytes aggravated TAC-induced LV functional impairment. Spatial transcriptomics of heart cryosections revealed a more pronounced inflammatory and pro-fibrotic phenotype in the inner, more stressed tissue niche of VDR^CM-KO^ compared with VDR^fl/fl^ mice after TAC.

Our finding that cardiomyocyte-specific VDR deletion does not result in LVH under basal conditions contrasts with the findings reported by Chen et al. [21]. The latter authors suggested that ablation of the VDR in cardiomyocytes results in LVH, both at baseline and after isoproterenol infusion in comparison to WT controls. Given that both studies employed the same Cre driver mouse line, the difference cannot be explained by specificity of Cre expression. The Cre driver mice used are a knock-in model in which parts of the *Mlc2v* gene were replaced by a Cre cassette [25]. In line with prior studies [26], our data show that *Mlc2v (Myl2)* haploinsufficiency does not result in a cardiac phenotype. In contrast, homozygous *Mlc2v* mutant mice develop a severe phenotype and die before birth [25]. The discrepancies between our findings and those of Chen et al. [21] might stem from the use of different VDR floxed mice: our model targets exon 3 [24], whereas theirs targets exon 4 of the *VDR* gene [21]. Nonetheless, both gene targeting strategies are expected to result in complete VDR inactivation. A more plausible explanation for the discrepancies could be differences in knockout approach: Chen et al. [21] used Mlc2v Cre-expressing VDR^fl/-^ mice as tissue-specific knockout model, i.e., VDR floxed mice on a heterozygous global VDR deficient background. This is a major difference from our study, and likely explains the more severe phenotype in their study [21] due to the interaction between cardiomyocyte-specific VDR deletion and global VDR haploinsufficiency. In light of our finding of an altered inflammatory cardiac phenotype in TAC mice lacking VDR in cardiomyocytes, VDR haploinsufficiency in immune cells may have a major modulating influence in these experiments.

In addition, we found germline deletion in our Mlc2v Cre model, a frequent problem encountered with Cre driver mice, which can make tissue-specific conditional knockout experiments uninterpretable [47]. Mating of Mlc2v Cre-positive VDR^fl/wt^ females with male VDR^fl/wt^ mice frequently resulted in germline VDR deletion. Germline deletion was not observed when male Cre-positive VDR^fl/wt^ mice were crossed with female VDR^fl/wt^ mice. Therefore, we solely utilized paternal inheritance of the Cre allele for the present study. Germline activation of Cre results in global deletion of the floxed allele. Hence, depending on the breeding strategy, this problem can result in heterozygous or even homozygous global deletion of the floxed gene, which in turn may bias the results.

Our finding that mice with a cardiomyocyte-specific deletion of the VDR exhibited more pronounced LV functional impairment after TAC than controls aligns with a post-hoc analysis of the EVITA (Effect of VITamin D on All-cause mortality in heart failure) trial. The latter trial investigated the impact of daily vitamin D supplementation for up to 3 years on various cardiac functional parameters in patients with advanced heart failure. Although the trial did not demonstrate any significant correlation between treatment duration and all examined functional parameters, a noteworthy improvement in cardiac function was noted in aged patients, indicated by a modest, yet statistically significant, increase in EF [48].

In our spatial transcriptomics analysis, we first applied the SPIN algorithm [29] to address autocorrelation issues between neighboring spots, enhancing the resolution of spatial patterns and improving cluster identification between hypertrophic and healthy heart tissues. This precision allowed for a detailed scrutiny of gene regulatory network (GRN). The analysis differentiated heart regions into clusters with distinct gene expressions indicative of normal heart function (outer regions) or stress-induced hypertrophy (inner regions). Notably, *Myh6* was prevalent in the outer, while *Myh7* was upregulated in the inner regions as adaptation to cardiac stress. In the inner region of the VDR^CM-KO^ TAC heart, a more inflammatory profile was evident compared to the VDR^fl/fl^ TAC heart, characterized by small regulatory differences and activation of unique complement and IL-9 pathways through de novo mechanistic GRNs driven by Mafg, Mafk, and Irf7.

VDR deficiency in the inner, stressed region also correlated with fibrosis-promoting regulatory networks. This finding is in agreement with the upregulation of *Col1a1* transcription assessed by qRT-PCR (Figure 2G). Hence, the more pronounced fibrosis-promoting regulatory networks in the inner region of VDR^CM-KO^ TAC hearts may translate into augmented cardiac fibrosis at later time points than the 4 weeks post-TAC used in the current study. CEBPB-driven GRNs appeared to be characteristic of the VDR^CM-KO^ TAC heart across the tissue niches. Yet, the specific genes targeted by these GRNs differed, likely reflecting the diverse microenvironmental influences. Given that CEBPB is influenced by the vitamin D pathway [49], the absence of VDR may explain its aberrant regulatory network. Conversely, the VDR^fl/fl^ mouse had more protective regulatory patterns in the inner, stressed region. We hypothesize that the LV functional impairment associated with VDR deficiency in cardiomyocytes after TAC is the consequence of the more inflammatory phenotype in the inner, stressed regions of the hypertrophic heart. This inflammatory response, coupled with fibrosis-driving GRNs, could potentially explain the observed exacerbation of HFrEF in VDR^CM-KO^ TAC mice. It is conceivable that the loss of VDR signaling in cardiomyocytes may lead to a more uncontrolled inflammatory response, which in turn may worsen cardiac function. It is interesting to note in this context that CEBPB expression in leukocytes has been linked to muscle function in observational studies [50], corroborating the association between inflammatory processes and muscle function.

A limitation of the current study is that we could compare only one heart cryosection of VDR^CM-KO^ and VDR^fl/fl^ TAC mice each in spatial transcriptomics analysis. Nevertheless, our study has uncovered that conditional ablation of the VDR in cardiomyocytes leads to a more pronounced pro-inflammatory and pro-fibrotic gene regulatory phenotype in the inner, more stressed tissue niche in the TAC-induced LVH model. Future studies will be needed to fully unravel the underlying molecular mechanisms of action. A better understanding of the interaction between vitamin D signaling and heart function may eventually lead to new treatment approaches in patients with heart failure.

## 4. Material and Methods

### 4.1. Animals

All animal procedures were approved by the Animal Welfare Committee of the Austrian Federal Ministry of Education, Science and Research and were undertaken in accordance with prevailing guidelines for animal care and welfare (BMWFW-68.205/0188-WF/V/3b/2017). All studies were carried out in male mice at 4–5 months of age. Genotype was assessed by PCR on genomic DNA isolated from mouse ear punches. Throughout the experiments, mice were housed at 24 °C with a 12 h light-dark cycle. They were fed a commercial rodent chow (Sniff, Soest, Germany), and had free access to food pellets and tap water. At necropsy, mice were euthanized by exsanguination from the abdominal vena cava under general anesthesia with ketamine/medetomidine (100/0.25 mg/kg) for serum collection.

### 4.2. Transverse Aortic Constriction (TAC)

The TAC procedure was performed to induce pressure overload and cardiac hypertrophy, as previously described [22]. Briefly, mice were anesthetized with ketamine/medetomidine (100/0.25 mg/kg i.p.), intubated, and ventilated using a small animal ventilator (Model 845, Harvard Apparatus, Holliston, MA, USA). The chest was opened via a midline sternotomy, and the transverse aorta was ligated using a 6-0 silk suture tied around a 27-gauge needle. The needle was then promptly removed, creating a stenosis in the aortic arch. Sham-operated animals underwent the same surgical procedure except that the ligature was not tied. After the TAC or sham procedure, the chest was closed in layers and the animals were allowed to recover on a warming pad. Buprenorphine (0.25 mg/kg s.c.) was administered subcutaneously every 24 h for four days to alleviate pain and enrofloxacin (10 mg/kg s.c.) for five days to prevent infection. Four weeks after the surgery, mice were sacrificed as stated above.

### 4.3. Doppler Echocardiography

Transthoracic in vivo echocardiography was performed to assess cardiac function using a linear transducer system (Accuson s2000tm, Siemens, Munich, Germany) equipped with a 14-MHz probe, as previously described [22]. Briefly, mice were anesthetized with 1.5% isoflurane and positioned in the supine position on a heating pad. M-mode images were obtained from the parasternal short-axis view at the level of the papillary muscles to measure left ventricular (LV) wall thickness and chamber dimensions (LVIDd, LVIDs, LVAW, and LVPW thickness). Fractional shortening (FS) and ejection fraction (EF) were calculated from these dimensions. At least five cardiac cycles were averaged for each measured parameter.

### 4.4. Central Arterial and Cardiac Pressure Measurements

Central arterial pressure was measured by inserting a SPR-671 micro-tip catheter (1.4 Millar Instruments, Houston, TX, USA) into the ascending aorta via the right carotid artery under 1.5% isoflurane anesthesia. The catheter was then further advanced into the left ventricle for cardiac pressure measurements. Pressure waveforms were recorded for at least three minutes after stable hemodynamic parameters were achieved and analyzed via LabChart7 software (V. 7.3.8, ADInstruments, Dunedin, New Zealand). Mean arterial blood pressure was calculated as 2/3 diastolic pressure plus 1/3 systolic blood pressure.

### 4.5. Biochemical Analysis

Serum levels of phosphate and calcium were measured using a Cobas c111 analyzer (Roche, Mannheim, Germany). Serum was extracted with diethylether and resuspended in steroid-free serum (DRG Diagnostics, Marburg, Germany) for the aldosterone ELISA (DRG Diagnostics).

### 4.6. Tissue Harvesting and Histological Analysis

Hearts were harvested and fixed in 4% formalin. Paraffin-embedded sections at 5-μm thickness were stained with FITC-conjugated wheat germ agglutinin (WGA) to evaluate cardiomyocyte size and picrosirius red (PSR) to assess collagen deposition and fibrosis in the heart as described previously [51]. Images were taken using a Zeiss LSM 880 Airyscan confocal microscope. Cardiomyocyte size and fibrotic areas were assessed with ImageJ (National Institutes of Health) with the investigator blinded for the experimental conditions.

### 4.7. RNA Isolation and Quantitative Real-Time PCR

Hearts were harvested from mice and snap-frozen in liquid nitrogen. Total RNA was extracted from heart tissue using a TRI Reagent solution (Applied Biosystems, Thermo Fischer Scientific, Waltham, MA, USA) as described previously [51,52]. The purity and integrity of the extracted RNA were assessed using electrophoresis (Agilent Tapestation, Santa Clara, CA, USA). Purified RNA (2 µg) was synthesized into cDNA using the High-Capacity cDNA Reverse Transcription Kit (Applied Biosystems, Thermo Fisher Scientific, USA). Quantitative RT-PCR was performed on the QTower device (Analytic Jena, Jena, Germany) using Fast Eva Green Kit (Biotium, Fremont, CA, USA) or by TaqMan probe, depending on the assay. All samples were measured in triplicate and normalized to either one reference gene (*Dpm1*) for *Fgf23*, *Col1a1,* and *Bnp* expression, or two reference genes (*Txnl4a* and *Dpm1*) for VDR expression. The 2^−ΔΔCt^ standard method was employed to calculate the relative expression level of genes.

### 4.8. Statistical Analysis of Phenotyping Data

All phenotyping data are expressed as mean ± standard error of the mean (SEM). Sample sizes were determined by analysis based on data collected by our laboratory in published studies [22,51,52]. Statistical analysis of the data was carried out using GraphPad Prism software (GraphPad Software 8.3.0, San Diego, CA, USA). Comparison between groups was made using one-way analysis of variance (ANOVA) followed by Student–Newman–Keuls test for multiple comparisons. Differences were considered statistically significant at values of *p* < 0.05.

### 4.9. Spatial Transcriptomics and Bioinformatic Analysis

Heart cryosections were air-dried, fixed in methanol at −20 °C, and stained with hematoxylin-eosin (HE) according to standard 10× Genomics protocols. Prior to hybridization, high resolution images of the HE-stained sections were taken. Spatial transcriptomics were performed according to standard procedures at the Genomics Core Facility of the Medical University Vienna, using the mouse 10× Genomics Fresh-frozen v2 kit on a CytAssist machine.

#### 4.9.1. Spatial Transcriptomics Data Preprocessing

Spatially resolved transcriptomic datasets from heart sections of TAC VDR^CM-KO^ and VDR^fl/fl^ mice were processed using Scanpy [53]. We performed quality control by filtering out spatial spots with low and extreme total RNA counts (<2500 and >20,000 counts) and genes detected in fewer than 20 spots (Supplementary Figure S4).

#### 4.9.2. Data Integration

The SPIN algorithm [29] integrated TAC VDR^CM-KO^ and VDR^fl/fl^ datasets, addressing batch effects while retaining spatial information. Dimensionality reduction via PCA and spatial pattern visualization through UMAP facilitated the identification of unique expression domains through Leiden clustering.

#### 4.9.3. Differential Expression and Marker Identification

We employed Scanpy’s differential expression analysis to find genes differentiating spatial tissue niches across VDR^CM-KO^ and VDR^fl/fl^ conditions. Selected markers were visualized across tissue sections, linking gene expression patterns to spatial regions.

#### 4.9.4. Functional Enrichment Analysis

GO (Gene Ontology) enrichment analysis, conducted with GSEApy [54] against different gene sets, such as comprehensive biological process, cellular component, and molecular function databases, elucidated the functional landscapes of spatially variable genes, identifying potential biological functions of observed spatial expression patterns.

#### 4.9.5. Differential Gene Regulatory Network Analysis

We conducted a comparative analysis using DiNiro [39] to examine the differences in gene regulatory networks between spots from the 1_KO region (region 1 in VDR^CM-KO^) and those from 1_fl (region 1 in VDR^fl/fl^). DiNiro is a computational tool designed to unravel regulatory mechanisms from single-cell data, providing valuable insights into gene expression patterns. Our analysis utilized the following parameters: number of subsamples = 10, sub-sampling size (%) = 80, occurrence threshold (%) = 100, significance cutoff = 0.05. For further details regarding parameter selection and their implications, we refer to the DiNiro publication [39].

#### 4.9.6. Co-Expression Modules and Gene-Regulatory Networks (GRN)

We employed SCANet [55] to investigate differences in gene co-expression networks (GCNs) among various spatial regions in the integrated dataset using the 6000 highly variable genes. SCANet inferred de novo GCN modules from the spatial data and analyzed region associations, identifying modules with altered expression in specific regions. These GCNs were further converted to gene regulatory networks (GRNs), and we conducted a drug repositioning analysis based on these networks.

## Data Availability

All mouse phenotyping data generated or analyzed in this study are included in this article (and its Supplementary Information Files). The spatial transcriptomics raw data that support the findings of this study are available at https://github.com/oubounyt/heart_visium (accessed on 23 May 2024).

## Supplementary Materials

The following supporting information can be downloaded at: https://www.mdpi.com/article/10.3390/ijms25115929/s1.
