# Supplementary material for: Ablation of Vitamin D Signaling in Cardiomyocytes Leads to Functional Impairment and Stimulation of Pro-Inflammatory and Pro-Fibrotic Gene Regulatory Networks in a Left Ventricular Hypertrophy Model in Mice
Source: Int J Mol Sci. 2024 May 29;25(11):5929. doi: 10.3390/ijms25115929 (PMC11172934; doi:10.3390/ijms25115929)
Supplement: Supplementary file 1 [file ijms-25-05929-s001.zip › ijms-2984807-supplementary.pdf]

Supplementary Material

Ablation of Vitamin D Signaling in Cardiomyocytes Leads to Functional Impairment and Stimulation of Pro-Inflammatory and Pro-Fibrotic Gene Regulatory Networks in a Left Ventricular Hypertrophy Model in Mice

Ana Zupcic 1,†, Nejla Latic 1,†, Mhaned Oubounyt 2,*, Alice Ramesova 1, Geert Carmeliet 3, Jan Baumbach 2, Maria L. Elkjaer 2,‡ and Reinhold G. Erben 1,4,*,‡

1 Department of Biomedical Sciences, University of Veterinary Medicine, 1210 Vienna, Austria; azupcic1@gmail.com (A.Z.); nejla.latic@vetmeduni.ac.at (N.L.); alice.ramesova@vetmeduni.ac.at (A.R.)

2 Institute for Computational Systems Biology, University of Hamburg, Albert-Einstein-Ring 8-10, 22761 Hamburg, Germany; jan.baumbach@uni-hamburg.de (J.B.); maria.louise.elkjaer@uni-hamburg.de (M.L.E.)

3 Department of Chronic Diseases, Metabolism and Ageing, 3000 Leuven, Belgium; geert.carmeliet@kuleuven.be

4 Ludwig Boltzmann Institute of Osteology, Heinrich-Collin-Strasse 30, 1140 Vienna, Austria

* Correspondence: mhaned.oubounyt@uni-hamburg.de (M.O.); reinhold.erben@osteologie.lbg.ac.at (R.G.E.)

† These authors contributed equally to this work.

‡ These authors contributed equally to this work.

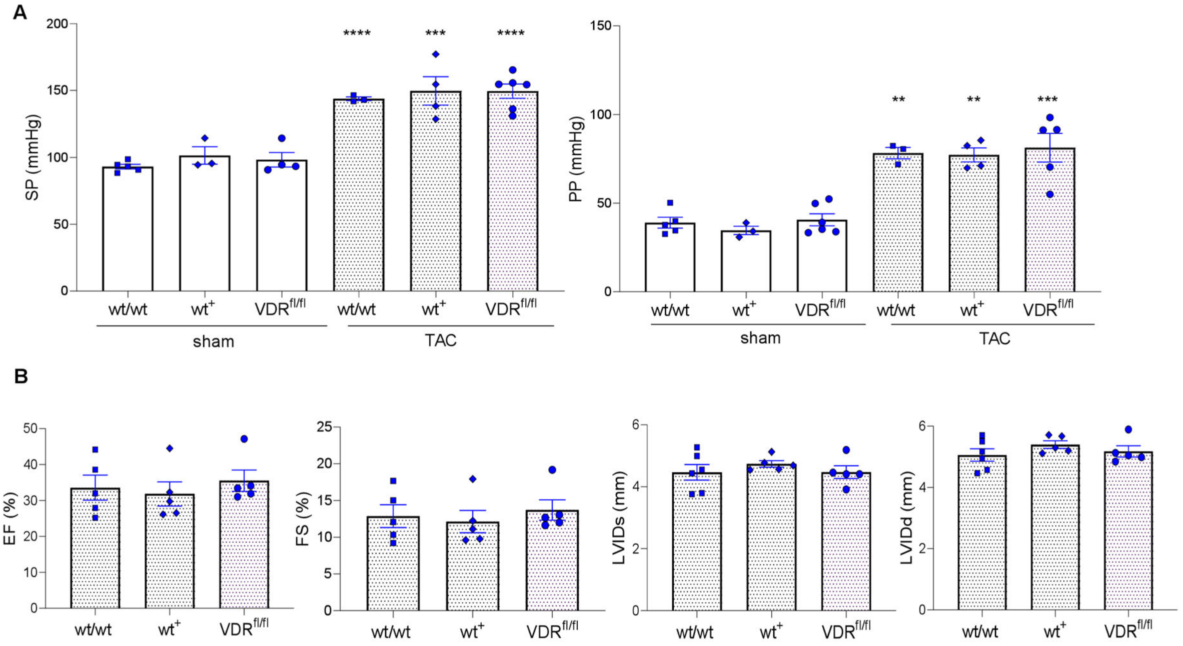

Supplementary Figure S1. **Cardiovascular phenotype of wt/wt, wt/Cre^{+} (wt^{+}), and VDR floxed (VDR$^{fl/fl}$) mice is comparable after TAC.** (**A**) Systolic pressure (SP) and pulse pressure (PP) are comparably increased in wt/wt, wt^{+} and VDR$^{fl/fl}$ mice following TAC (n=5 wt/wt sham; n=3 wt^{+} sham; n=4 VDR$^{fl/fl}$ sham for SP and n=6 for PP; n=3 wt/wt TAC; n=4 wt^{+} TAC; n=6 VDR$^{fl/fl}$ TAC for SP and n=5 for PP). (**B**) Ejection fraction (EF), fractional shortening (FS) (n=5 wt/wt TAC; n=5 wt^{+} TAC; n=5 VDR$^{fl/fl}$ TAC), as well as left ventricular internal diameter in diastole (LVIDd) and systole (LVIDs) (n=6 wt/wt TAC; n=5 wt^{+} TAC; n=5 VDR$^{fl/fl}$ TAC) do not show differences between TAC wt/wt, wt^{+} and VDR$^{fl/fl}$ mice. Data are given as bar dot plots with SEM. ** $p < 0.01$, *** $p < 0.001$, **** $p < 0.0001$ by one-way ANOVA followed by Student-Newman-Keuls post-hoc test.

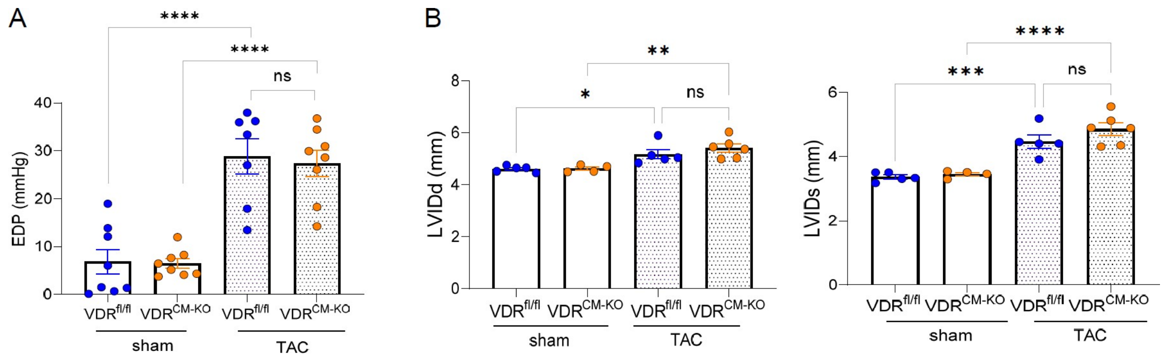

Supplementary Figure S2. **Mice lacking VDR in cardiomyocytes and VDR$^{fl/fl}$ control mice show comparable increases in end-diastolic pressure and left ventricular internal diameter after TAC.** (**A**) End-diastolic pressure (EDP) measured by left ventricular catheterization is comparably increased in VDR$^{fl/fl}$ and VDR$^{CM\text{-}KO}$ mice following TAC (n=8 VDR$^{fl/fl}$ sham; n=8 VDR$^{CM\text{-}KO}$ sham; n=7 VDR$^{fl/fl}$ TAC; n=8 VDR$^{CM\text{-}KO}$ TAC). (**B**) Left ventricular internal diameter in diastole (LVIDd) and systole (LVIDs) measured by echocardiography are increased in TAC VDR$^{CM\text{-}KO}$ and VDR$^{fl/fl}$ mice, relative to sham-operated controls (n=5 VDR$^{fl/fl}$ sham; n=4 VDR$^{CM\text{-}KO}$ sham; n=5 VDR$^{fl/fl}$ TAC; n=6 VDR$^{CM\text{-}KO}$ TAC). Data are given as bar dot plots with SEM. * $p < 0.05$, ** $p < 0.01$, *** $p < 0.001$, **** $p < 0.0001$ by one-way ANOVA followed by Student-Newman-Keuls post-hoc test.

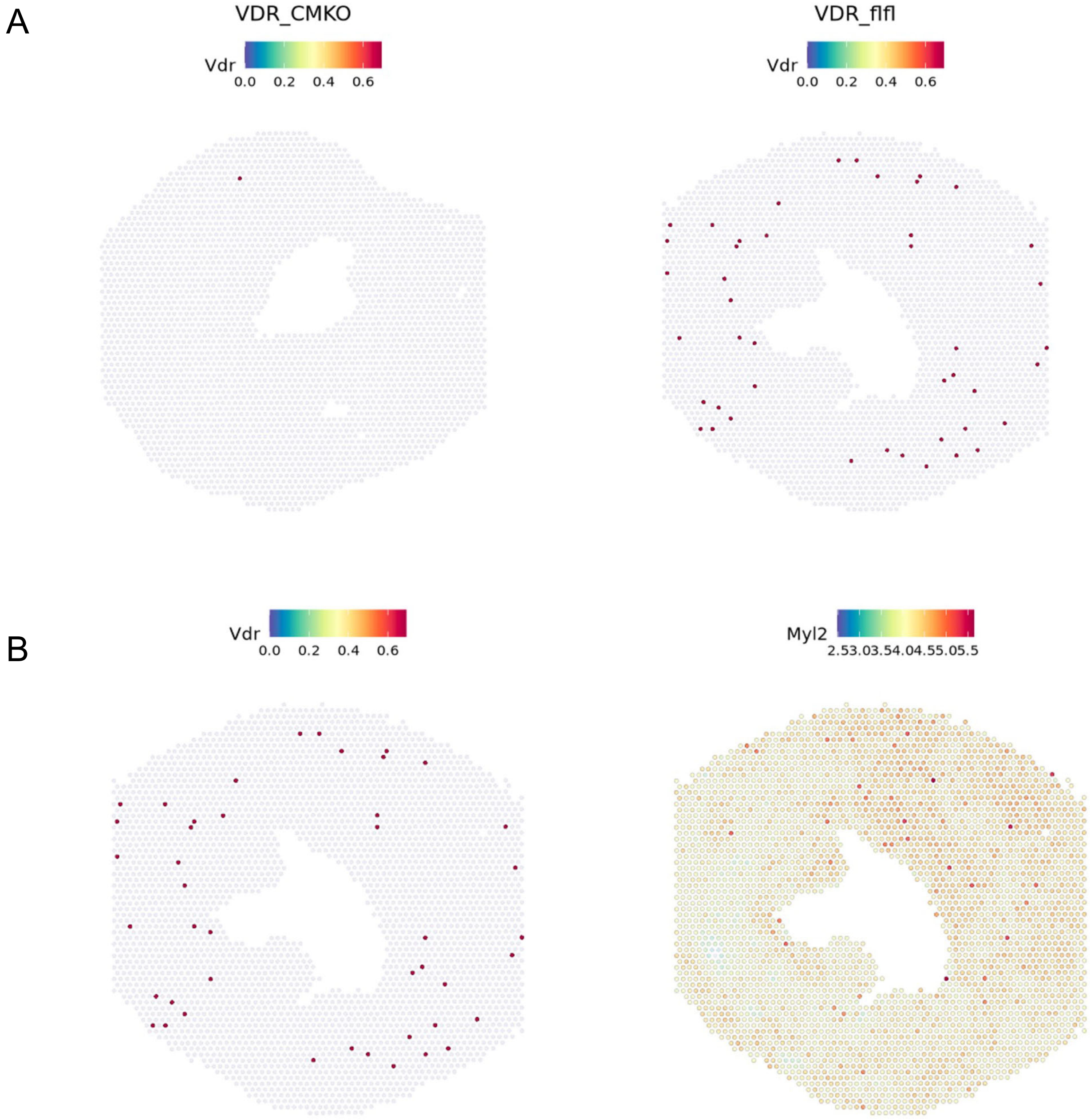

Supplementary Figure S3. Cardiac VDR expression assessed by spatial transcriptomics. (A) Representation of the spatial distribution of VDR expression in heart cross-sections of $VDR^{CM\text{-}KO}$ and $VDR^{fl/fl}$ TAC mice. **(B)** Colocalization of VDR and the *Myl2* gene within heart tissue of the $VDR^{fl/fl}$ mouse post-TAC. *Myl2* was present in all spots where VDR was expressed (a total of 44 spots).

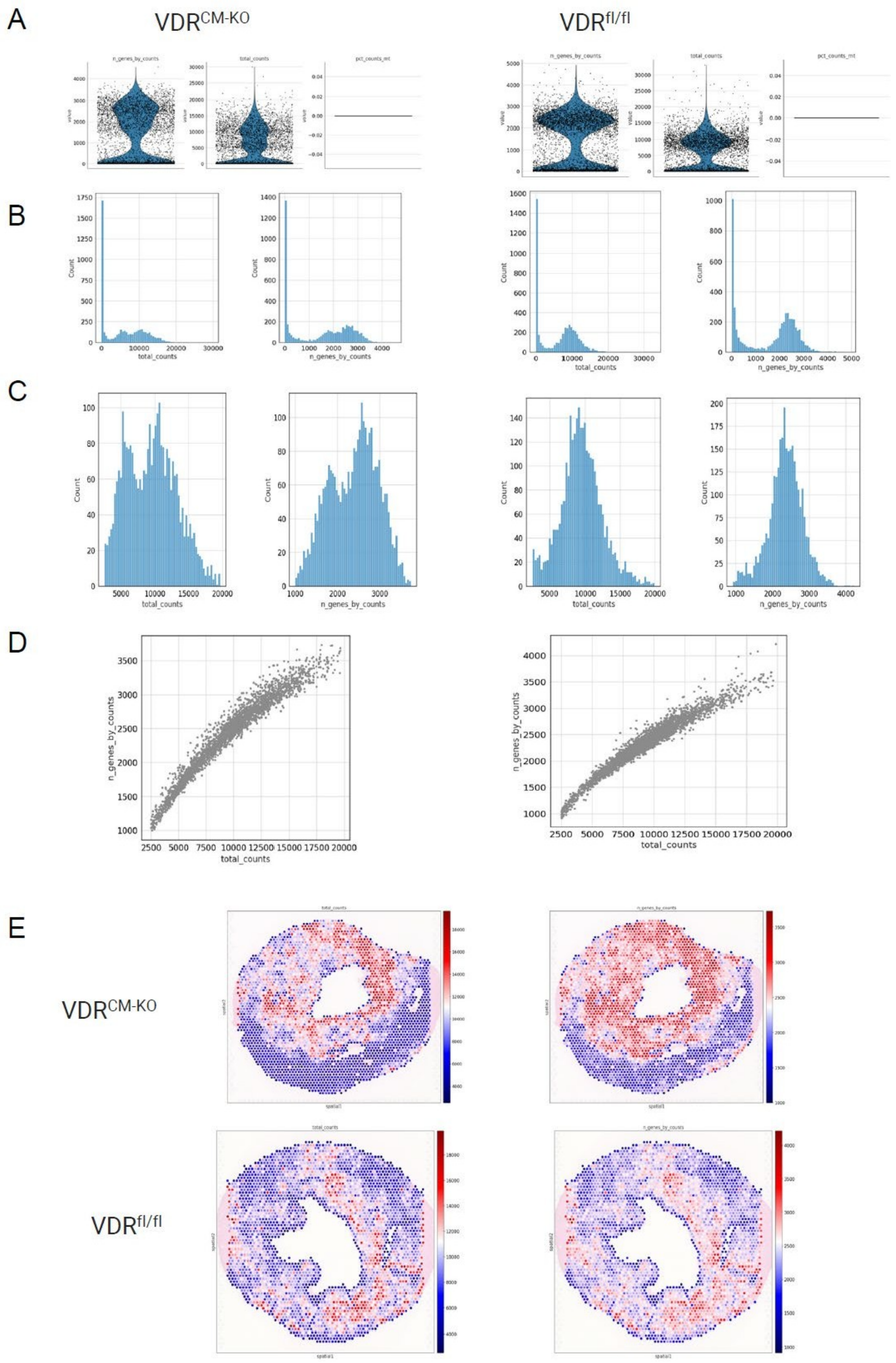

Supplementary Figure S4. Quality Control (QC) and preprocessing analysis of the spatial transcriptomics data using Scanpy QC metrics. (**A**) Plots illustrating the distribution of expressed genes and total counts per spot, alongside the percentage of mitochondrial genes in VDR$^{fl/fl}$ and VDR$^{CM\text{-}KO}$ samples. (**B**) Histogram showing the distribution of total counts and of expressed genes before filtering. (**C**) Filtering steps involved removing low-quality spots with less than 2,500 counts and those with more than 20,000 counts, as well as genes expressed in fewer than 20 spots. (**D**) Scatter plot depicting total counts (X-axis) versus the number of genes (Y-axis) after filtering. (**E**) Visualization of the number of expressed genes and total counts after QC filtering on the spatial coordinates.

Supplementary Table S1. Primer sequences used for quantitative real-time PCR analysis.

**Gene**	**Forward (5´- 3´)**	**Reverse (5´- 3´)**
Bnp	GCC AGT CTC CAG AGC AAT TCA	GCC ATT TCC TCC GAC TTT TCT
Col1a1	CCG GCT CCT GCT CCT CTTA	CCA TTG TGT ATG CAG CTG AC TTC
Dpm1	AGC GTC AGA CTT AAC AGG AAG CT	TAA CCC TTT CAG GAA CGA GAC TA
Txnl4a	TGC ATG AAG ATG GAC GAG GTT	CAA GAT GAG GAA GAT CTC CCA CT

**Gene**	**Forward (5´- 3´)**	**Probe**	**Reverse (5´- 3´)**
Vdr	GGC TTC CAC TTC AAC GCT ATG	56-FAM/CCTGTGAAG/ZEN/GCT GCAAGGGTTTCTTCA/3IAB-kFQ	GGG CCT TGC GCT TCATG
Fgf23	TAGGGATGGTC ATGTAGATGGC A	56-FAM/CCCCATCAG/ZEN/AC-CATCTACAGTGCCCTG/3IAB-kFQ	ATG GCT CCT GTT ATC ACC ACAGA
